# Clinical analysis of orthostatic headache in Korean patients

**DOI:** 10.1186/1129-2377-14-S1-P153

**Published:** 2013-02-21

**Authors:** YJ Choi, BH Cho, MH Park, TS Nam, JT Kim, SH Lee, BC Kim, MK Kim, KH Cho

**Affiliations:** 1 Chonnam National University Hospital, Korea, Republic of China; 2 Chonnam National University Hwasun Hospital, Korea, Republic of China

## Introduction

Orthostatic headache is defined as a headache that is significantly worsened in the standing position and relieved with recumbency. The major cause of orthostatic headaches is cerebrospinal fluid (CSF) leak. But orthostatic headache also occurs in variable diseases. Therefore clinical manifestations, MRI findings, and outcomes are seems to be various.

## Objectives

We analyzed clinical and radiological differences between favorable and non-favorable groups.

## Methods

We reviewed 45 patients with orthostatic headache. All patients were underwent brain MRI. CSF tapping and radioisotope cisternography was performed as occasion demands. All patients were performed conservative therapy. Autologous blood patch was done in patients who did not respond to conservative therapy. We divided patients to two groups, favorable (F), unfavorable (UF) groups. The F group defined as clinical improvement by conservative therapy, or once trial of blood patch. More than 14 days of hospitalization, two more trials of blood patch, or relapse defined as UF group.

## Results

21 of 45 patients were classified as a F group. There were no significant differences in age between two groups. The F group had short hospitalization period (7.5 days vs 16.0 days, p=0.009). The UF group had more abnormal MR findings (5 vs 17, p<0.001). There were 1 platybasia, 1 skull base tumor, 1 Chiari I malformation, 1 enhancement of dural and epidural layer of thoracic spine, 3 pituitary enlargement, 3 sagging brain and 4 subdural hemorrhages in UF group. 12 of UF group showed pachymeningeal enhancement in brain MRI (3 patients in F group, p<0.001). 16 of F group and 7 of UF group showed normal MRI. 1 of 16 normal MRI in F group and 5 of 7 normal MRI in UF group had CSF leakage on non-lumbar lesion (p<0.001).

## Conclusions

Orthostatic headache presenting unfavorable outcome had more brain MRI abnormalities and CSF leakage on upper spinal level. Due to small in number of cases, further recruit of patients is needed.
